# An Indian national survey of therapeutic drug monitoring with anti-tumor necrosis (TNF) medications in inflammatory bowel disease

**DOI:** 10.1007/s12664-020-01047-6

**Published:** 2020-06-01

**Authors:** Rajan N. Patel, Gaurav B. Nigam, Raj G. Jatale, Devendra Desai, Govind Makharia, Vineet Ahuja, Jimmy K. Limdi

**Affiliations:** 1 The Whittington Hospital NHS Trust, London, UK; 2 The Pennine Acute Hospitals NHS Trust, Manchester, UK; 3 Department of Bio-Statistics, P D Hinduja Hospital, Mumbai, 400 016 India; 4 P D Hinduja Hospital, Mumbai, 400 016 India; 5 The All India Institute of Medical Sciences, New Delhi, 110 029 India; 6 The Pennine Acute Hospitals NHS Trust, Manchester Academic Health Sciences, University of Manchester, Manchester, UK

**Keywords:** Adalimumab, Crohn’s disease, Drug levels, Infliximab, Pharmacokinetics, Ulcerative colitis

## Abstract

**Background:**

Evidence supports therapeutic drug monitoring (TDM) in improving efficacy and cost-effectiveness of anti-TNF therapy in inflammatory bowel disease (IBD). Data on perceptions and barriers to TDM use are limited and no data are available from India. Our objective was to assess clinicians’ attitudes and barriers to TDM use in IBD.

**Methods:**

A 16-question survey was distributed to members of the Indian Society of Gastroenterology. Information on clinician characteristics, demographics, use and barriers towards TDM with anti-TNFs was collected. Logistic regression was used to predict factors influencing TDM use.

**Results:**

Two hundred and forty-two respondents participated (92.5% male); 83% were consultant gastroenterologists. Of 104 respondents meeting inclusion criteria (treating > 5 IBD patients and at least 1 with an anti-TNF per month), complete responses were available for 101 participants. TDM was utilized by 20% (*n* = 20) of respondents. Of them, 89.5% (*n* = 17) used TDM for secondary loss of response; 73.7% (*n* = 14) for primary non-response and 5.3% (*n* = 1) proactively. Barriers to TDM use were cost (71.2%), availability (67.8%), time lag in results (58.7%) and the perception that TDM is time-consuming (45.7%). Clinicians treating > 30 IBD patients were more likely to check TDM (OR = 4.9, *p* = 0.02). Of 81 respondents not using TDM, 97.5% (*n* = 79) would do so if all the barriers were removed.

**Conclusion:**

Significant barriers to TDM use were availability, cost and time lag for results. If these barriers were removed, almost all the clinicians would use TDM at least reactively and 25% would use proactively. There is an urgent need to address these barriers and optimize anti-TNF therapy for optimal outcomes.

**Electronic supplementary material:**

The online version of this article (10.1007/s12664-020-01047-6) contains supplementary material, which is available to authorized users.

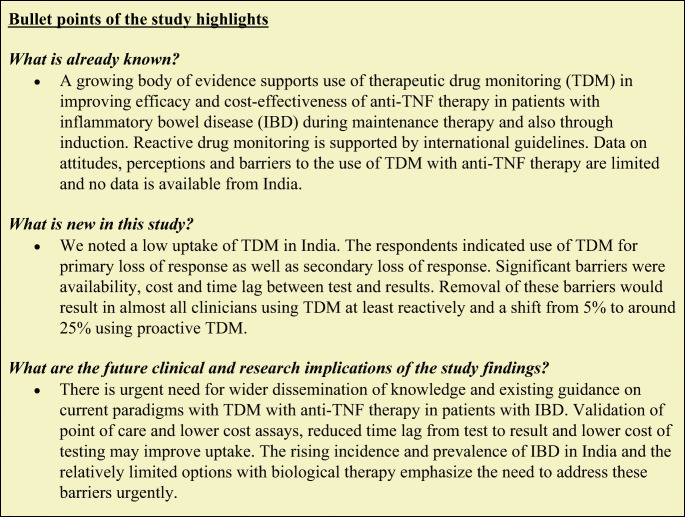



## Introduction

Anti-TNF therapies have transformed the care of patients with inflammatory bowel disease (IBD). They have re-defined our perceptions around meaningful disease control - moving beyond symptom control to bolder definitions such as mucosal healing, histological and deep remission and an improvement in quality of life [1, 2]. “Treating to target” and achieving mucosal healing when possible is now an important priority [3–5]. Gastroenterologists currently have relatively limited options to achieve this. To contextualize, in India, the mainstay of biological therapy currently is anti-TNF therapy with anti-integrin therapy becoming available soon.

Anti-TNF therapies are immunogenic and are associated with loss of response [6]. Up to 30% of patients have a primary non-response (PNR) and up to 50% will develop a secondary loss of response (SLR) to anti-TNFs [7, 8]. This can be caused by low or undetectable drug concentrations due to immune (anti-drug antibodies) and non-immune clearance [7, 9]. The risk of attrition can make each successive therapy less effective, implying that the first biologic is often most likely to be the most effective. Meanwhile, aiming for higher anti-TNF trough levels may be associated with better outcomes during both maintenance [10–18] and induction [19–22].

Cost of biological therapy has also posed limitations to their use but the approval of biosimilars for infliximab and adalimumab may serve to broaden the reach of these highly effective therapies [23, 24]. Taken together, this emphasizes the need to select wisely and optimize anti-TNF therapy in well-selected patients.

Therapeutic drug monitoring (TDM) involves measuring serum drug trough concentrations and anti-drug antibodies to optimize the use of anti-TNF agents [25–28]. TDM can be either reactive or proactive. Reactive TDM involves checking serum drug trough levels and anti-drug antibodies when there is a suspicion of loss of response to anti-TNF therapy [10–18, 25]. It has been shown to be cost-effective compared to empiric dose escalation [29–31]. Conversely, proactive TDM involves checking serum drug trough levels and anti-drug antibodies at pre-determined time points, irrespective of disease activity, with the aim of preventing “under-dosing” from triggering a disease flare and de-escalating dosing in case of “supra-therapeutic” drug levels [19–22, 25]. The use of TDM, at least reactively, is supported by international IBD guidelines [26–28, 32–34]. Data on attitudes, perceptions and barriers to the use of TDM with anti-TNF therapy are scarce, limited to two studies, from the USA and the UK respectively [35, 36]. India has the second largest IBD population in the world but limited access to biological choices compared with the Western world, making a compelling argument for optimizing the therapies available, through the use of TDM.

We conducted a survey on the use of TDM with anti-TNF therapy in India. Our primary aim was to describe the proportion of gastroenterologists utilizing TDM, the clinical setting in which this was used and to identify barriers to the use of TDM in clinical practice. Our secondary aim was to identify the clinical scenarios in which TDM would be used by gastroenterologists if all perceived barriers to TDM were removed.

## Methods

### Study design

A 16-question survey (Appendix 1) was adapted with permission from a similar study conducted in the UK [36]. The survey underwent a second modifying process in consultation with gastroenterologists (DD, GM and VA) at two large centres in India to ensure its suitability for participants. This was then placed on an online survey tool and an invitation with a link to complete the same was sent out to consultants and higher specialist trainee (Registrar/Fellow) members of the Indian Society of Gastroenterology (ISG) (approximately 1500 members) between June and October 2019. TDM was performed at either All India Institute of Medical Sciences (AIIMS), New Delhi or P D Hinduja Hospital, Mumbai. The study was registered with and approved by the Research and Innovation department of the Pennine Acute Hospitals NHS Trust, UK. No funding was required for this study.

Demographic information sought from the participants included their age, sex, grade (consultant, gastroenterology trainee/registrar, physicians with special interest in gastroenterology), number of years in practice since specialist qualification or accreditation for gastroenterology (as applicable), place of work (government medical college, private medical college, private group practice/corporate hospital or private individual practice) and city of work (tier 1/Metropolitan, tier 2/other state capitals or tier 3/all other cities). Additionally, information was collected from respondents regarding the proportion of patients with IBD seen in their clinical practice, number of patients with IBD treated personally in a 1-month period and numbers treated with anti-TNF therapy per month. We also sought details around the use of TDM using Likert 5-point scales ranging from strongly agree to strongly disagree, to identify levels of agreement or disagreement with potential barriers to using TDM. Participants who treated < 5 IBD patient per month and/or had no patients on anti-TNF therapy every month were excluded from the study.

### Statistical analysis

The data were analyzed using R software Version 3.5.2 (R development core team, Vienna, Austria). All variables were categorical and expressed as frequencies and percentages. Univariate logistic regressions were used to examine associations between available variables and the outcomes of interest, use of TDM and proactive TDM. Associations were reported as *p*-values and odds ratios, along with their 95% confidence intervals. To determine the independent effects of variables associated with the use of reactive and proactive TDM, a multiple binary logistic regression analysis was then performed including variables with a *p*-value of < 0.1 from univariate analysis.

## Results

Responses were received from 242 participants, of whom 104 met inclusion criteria (138 clinicians reported treating less than 5 IBD patients per month and/or having no patient on anti-TNF therapy and were therefore excluded). Baseline characteristics of all the participants are depicted in Table 1.

**Table 1:** Participant demographic and clinical characteristics.  *GI* gastrointestinal, *IBD* inflammatory bowel disease

Participants	*N* = 242
Gender	
Male	223 (92.2%)
Female	19 (7.8%)
Practice setting (more than one)	
Government medical college	56 (23.1%)
Private medical college	29 (12%)
Private group practice/corporate hospital	92 (38%)
Private individual practice	81 (33.5%)
Grade	
Consultant gastroenterologist	201 (83.1%)
Physician with special interest in GI practice	15 (6.2%)
Gastroenterology trainee	18 (7.4%)
Other	8 (3.3%)
City of practice	
Tier 1 (Metropolitan)	109 (45%)
Tier 2 (other state capitals)	47 (19.4%)
Tier 3 (all other cities and towns)	86 (35.6%)
Age (year)	
25–34	45 (18.6%)
35–44	82 (33.9%)
45–54	46 (19%)
55–64	53 (21.9%)
> 65	16 (6.6%)
Years (post gastroenterology certification) in practice/ Still in training	
< 1	15 (6.2%)
1–4	14 (5.8%)
5–9	48 (19.8%)
10–19	43 (17.8%)
> 20	46 (19%) 76 (31.4%)
% of patients with IBD in individual practice, (238 responses, 4 skipped)	
< 10%	180 (75.6%)
11–25%	56 (23.5%)
26–50%	2 (0.9%)
> 50%	0 (0%)

**Fig. 1:**
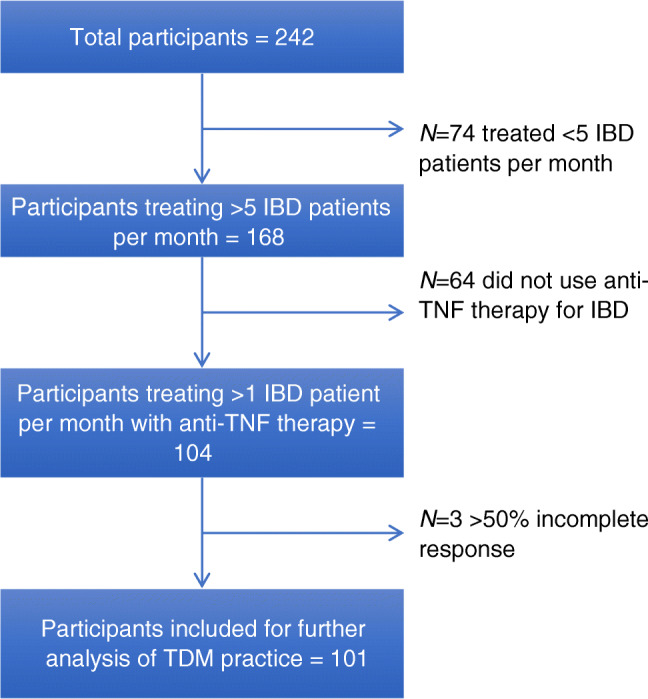
Flow diagram showing inclusion of participants for further analysis. *IBD* inflammatory bowel disease, *TNF* tumor necrosis factor, *TDM* therapeutic drug monitoring

Based on the inclusion criteria, only 104 participants were included for further analysis. This is represented as a flow diagram in Fig. 1 and the details are included in Table 2.

**Table 2:** Participant characteristics based on inclusion criteria. *IBD* inflammatory bowel disease, *TNF* tumor necrosis factor

Number of patients with IBD treated per month, (168 responses)	
5–10	81 (34.3%)
11-20	39 (16.5%)
20-30	27 (11.4%)
> 30	21 (8.9%)
No. of patients with IBD treated with anti-TNF per month, (104 responses)	
1–4	94 (56.3%)
5-10	10 (6%)
11-20	0 (0%)

### Practice of TDM

**Fig. 2:**
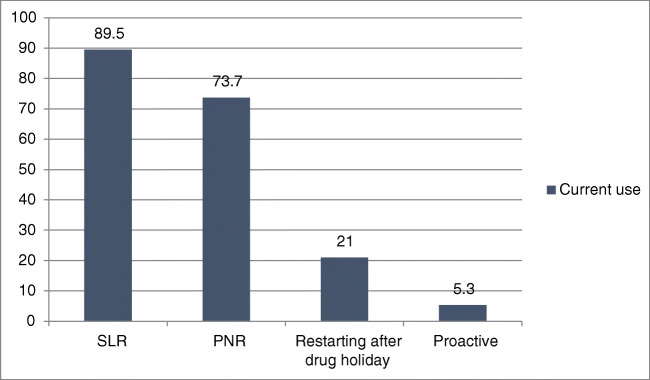
Current use of therapeutic drug monitoring. *PNR* primary non-response, *SLR* secondary loss of response

Of the 104 participants included in this analysis, completed responses were available for only 101 participants. TDM was utilized in clinical practice by 20% (*n* = 20) of respondents. Of them, 89.5% (*n* = 17) used TDM for SLR; 73.7% (*n* = 14) used it for PNR; 21% (*n* = 4) used it before restarting anti-TNF therapy after a drug holiday; and 5.3% (*n* = 1) used TDM proactively (Fig. 2). Of the 242 initial respondents, 64 clinicians (26.44%) reported not using biological therapies at all to manage their patients with IBD.

Multivariate analysis identified practice in tier 2 cities (*p* = 0.047), having 11% to 25% patients with IBD in their practice (*p* = 0.018) and seeing/treating a higher number of IBD patients per month (*p* = 0.036) as factors independently associated with the use of TDM (Table 3).

**Table 3:** Univariate and multivariate logistic regression analysis of variables associated with the use of therapeutic drug monitoring. *GI* gastrointestinal, *IBD* inflammatory bowel disease, *TNF* tumor necrosis factor, *OR* odds ratio, *CI* confidence interval

Variables		Univariate analysis			Multivariate analysis		
		*p*-value	OR	95% CI	*p*-value	OR	95% CI
Practice setting		0.890					
	Government medical college		1				
	Private medical college	0.800					
	Private group practice/corporate hospital	0.604					
	Private individual practice	0.601					
City of practice		0.098					
	Tier 1 (Metropolitan)		1			1	
	Tier 2 (other state capitals)	0.084			*0.047*	0.1	0.01–0.97
	Tier 3 (all other cities and towns)	0.634				0.6	
Grade		0.176					
	Consultant gastroenterologist		1				
	Physician with special interest in GI	0.999					
	Gastroenterology trainee	0.998					
	Others/surgeon	0.999					
Gender		0.148					
	Female		1				
	Male	0.132					
Age Group		0.171					
	25–34 years		1				
	35–44 years	0.058					
	45–54 years	0.234					
	55–64 years	0.199					
	> 65	0.998					
Years of practice		0.249					
	Still in training		1				
	< 1 years	0.273					
	1–4 years	0.919					
	5–9 years	0.288					
	10–19 years	0.728					
	> 20 years	0.552					
Percentage of patients with IBD		*0.009*					
	< 10		1				
	11–25%	*0.004*	4.8	1.7–13.9	*0.018*	3.9	1.3–12.4
	26–50%	0.998					
No. of patients with IBD reviewed/treated per month		*0.035*					
	5–10		1			1	
	11–20	0.686			0.745		
	21–30	0.154			0.261		
	> 30	*0.021*	4.9	1.3–20	*0.036*	3.5	0.8–16.1
No. of patients with IBD on anti-TNF therapy per month		0.282					
	0		1				
	1–4	0.890					
	5–10	0.214					

**Fig. 3:**
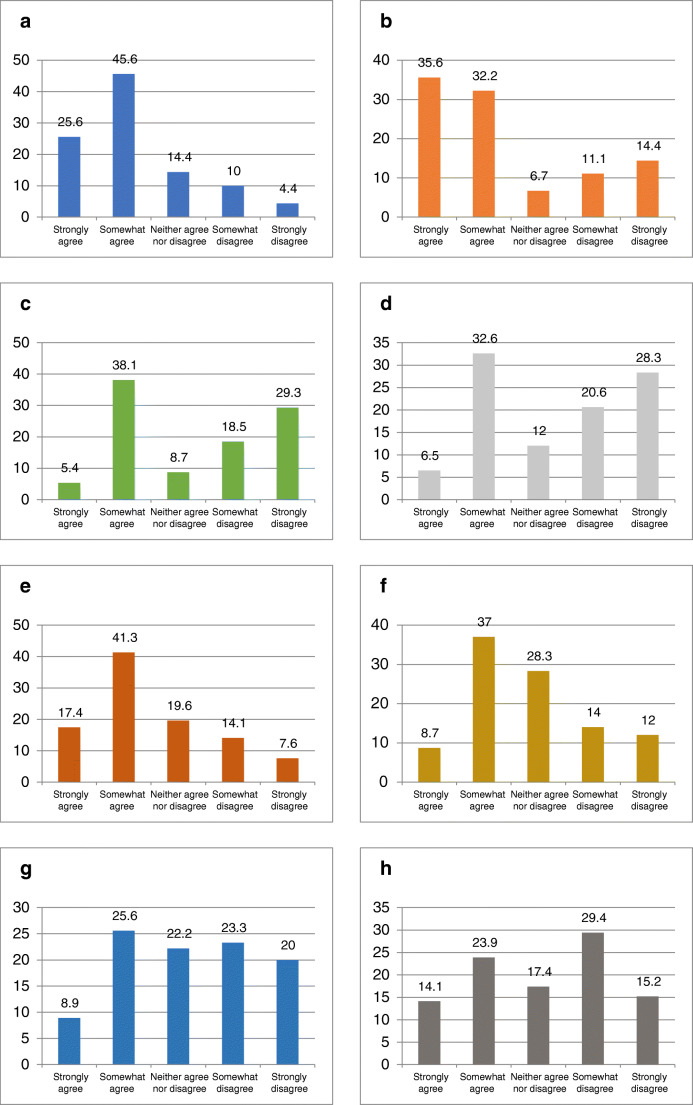
**a** Barriers to therapeutic drug monitoring (TDM): test is expensive; **b** uncertainty about availability in my practice; **c** lack of overall knowledge of TDM; **d** lack of knowledge on how to interpret and what to do with the results of TDM; **e** time lag from serum sampling to results of TDM; **f** TDM is cumbersome and/or time-consuming; **g** lack of good evidence-based medicine of the usefulness of TDM in inflammatory bowel disease; **h** lack of clinical guidelines recommending the use of TDM

The main barriers for TDM use reported by the respondents were cost (71.2%), uncertainty about availability (67.8%), time lag in receiving results (58.7%) and the perception that TDM is cumbersome and time consuming (45.7%). Respondents mostly disagreed or strongly disagreed that lack of overall knowledge of TDM (47.8%), lack of knowledge regarding how to interpret TDM and what to do with results of TDM (48.9%), lack of awareness of clinical guidelines (44.6%) and perceived lack of an evidence base for TDM use (43.3%) were barriers to its use (Fig. 3).

**Fig. 4:**
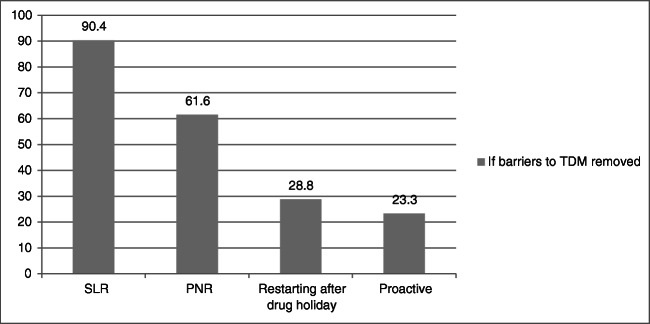
If barriers to therapeutic drug monitoring removed. *PNR* primary non-response, *SLR* secondary loss of response, *TDM* therapeutic drug monitoring

If all the barriers to TDM use were removed, 79 out of the 81 respondents currently not practising TDM would perform it more frequently. Amongst them, 90.4% would check TDM for SLR, 61.6% for PNR, 28.8% when restarting after a drug holiday and 23.3% would check it proactively (Fig. 4). 79.3% of these would check TDM proactively at least once a year if all barriers were removed.

## Discussion

This is the first National Survey of TDM use amongst clinicians treating IBD in India and only the third such survey in the world; so far, only two studies, one from the USA and the other from UK, has been published, underscoring the general lack of information on clinicians’ attitudes, perceptions and barriers to the use of anti-TNF TDM [35, 36].

Despite an increasing therapeutic armamentarium to treat IBD including biologicals and small molecule, treatment options in IBD remain largely limited as compared to other immune-mediated diseases. Moreover, anti-TNF therapies are currently the only available biological treatment option in India, with anti-integrin therapy (VDZ) likely to become available imminently. Progressive nature of IBD and the risk of attrition of response when changing from one treatment to another emphasize the need to optimize therapy before changing drugs within or outside class. Substantial variation in anti-TNF drug exposure and response to treatment underscores the importance of treatment optimization based on TDM. Consequently, TDM has emerged as the new standard of care for optimizing anti-TNF therapy in IBD, with reactive TDM being endorsed for assessment of PNR and SLR by recent international guidelines [26–28, 32–34]. Despite this, its use in clinical practice, since it first became available for use in 2016 in India, has not been assessed. 

We found that only 20% of respondents reported using TDM in their practice. This is in contrast to 90.1% and 96.6% in the USA and UK studies, respectively [35, 36]. Of those respondents using TDM, 89.5% used it to assess for secondary loss of response and 73.7% for primary non-response. These figures are comparable with the recent outcomes from a Western population of IBD clinicians (96% and 72% in UK, 87% and 66% in USA).

Working in smaller (tier 2) cities, having between 11% and 25% of one’s practice made up of IBD patients and seeing/treating a higher number of IBD patients per month were factors independently associated with using TDM. This suggests that clinicians working in more manageable environments (lower overall population of patients and lower burden of IBD) who are able to follow up their IBD patients more frequently are more likely to use TDM. This contrasts to the UK study, which identified an association between clinicians having a larger IBD patient population (> 50% of their practice) and TDM use [36].

Only 5.3% (*n* = 1) (compared with 54% and 36.6% in UK and US surveys, respectively) used TDM proactively [35, 36]. Asia Pacific guidance on the use of biologics supports reactive TDM in patients with active IBD to help guide management [37]. However, there is a growing body of evidence supporting proactive TDM during maintenance treatment [19, 21, 22, 38].

We assessed the predominant barriers to the use of TDM amongst our respondents and found these to be cost (71.2%), availability (67.8%), time lag from serum sampling to results (58.7%) and “cumbersome” nature of performing TDM (45.7%). Time lag to results was the only barrier common to clinicians working in UK, USA and India [35, 36]. In addition to the cost of the biologic agent, which is approximately INR 18,000 (biosimilar) to INR 24,000 (originator) per month, for a 60-kg person during maintenance, the cost of TDM is INR 14,000 for measurement of both drug levels and drug antibodies. Respondents may also be deliberating the need for repeated monitoring when considering cost as a barrier to TDM use. From our respondents not using TDM, if all barriers were removed, 97.5% would start using it as an IBD management strategy. Of them, most would use it reactively but up to one quarter would use TDM proactively. Therefore, a more affordable ‘point of care’ assay would enable wider adoption of TDM-based treatment optimization.

Lack of knowledge, awareness of guidelines and evidence were not barriers to the use of TDM in the majority of respondents from India. This probably highlights the fact that IBD care is a niche area practiced by clinicians with a special interest. Presumably, these clinicians are well aware of current guidelines on the management of IBD patients. Notably, a study exploring understanding and interpretation of TDM using TDM-based clinical scenarios demonstrated marked heterogeneity in its practical use, understanding and interpretation [39]. This makes sense when one acknowledges that TDM is a relatively newer concept, albeit integrated through evidence into standard of care, and that its use may still be limited by experience and awareness of various assays and the heterogeneity therein. It also makes a compelling case for a more robust approach through multidisciplinary care provided by experienced IBD clinicians and an unmet training need.

Additionally, the potential for population pharmacokinetics to identify parameters and sources of variability with dosing may enable clinicians to apply individual dosing schedules using a dashboard system to calculate the exact dose a patient should receive and at what time to maintain optimal drug concentrations [40, 41]. Meanwhile, ‘point of care’ assays may be able to rapidly measure trough concentrations enabling efficacy through speedy and accurate dose optimization [25, 42]. Reassuringly, TDM has been shown to be cost-effective compared to empiric dose escalation [29–31].

A significant strength of our survey is the involvement of respondents across different practice settings and experience levels in India. Despite our wide reach through the ISG membership, we acknowledge the possibility of a selection bias, which may apply to most survey-based studies [35, 36]. We were limited by the overall number of IBD patients treated by individual clinicians. This resulted in a significant number of respondents being excluded from the study, a small number of respondents looking after > 25% IBD patients within their practice and few treating more than 5 patients per month with an anti-TNF. Consequently, our sample size was small.

Inconsistencies with the use of TDM for anti-TNF therapy, despite international guidelines endorsing their applicability for optimizing therapy, are an important area of unmet need, which should be addressed through educational initiatives, seminars and publication with wide access to practising gastroenterologists [26–28, 32–34, 39–41]. Collaborative working, discussion within IBD multidisciplinary teams and access to more specialized units will promote best practice and achieve more optimal patient outcomes.

In conclusion, we found that only 1 in 5 surveyed gastroenterologists in India are using TDM within their IBD practice. Significant barriers were availability, cost and time lag between test and results. Removal of these barriers would result in almost all clinicians using TDM at least reactively and a shift from 5% to around 25% using proactive TDM. The development of low-cost assays would inevitably result in a surge in TDM use paralleling the effect that biosimilars have had in increasing biologic use in the West. Meanwhile, dashboard systems and novel approaches using population pharmacokinetics may serve to optimize drug exposure through predictive modelling.

The rising incidence and prevalence of IBD in India, coupled with increasing complexity of disease phenotypes and availability of biosimilar versions of anti-TNF, will improve access to therapy making a compelling argument to optimize available therapies to enable best possible patient outcomes. The real-world impact of these rapid strides and the altruistic pursuit of meaningful targets, however, hinges on the wider adoption of treatment optimization in practice and, in doing so, exemplifying the virtues of personalized medicine.

## Electronic supplementary material

ESM 1 (PDF 57 kb)
