# Supplementary material for: An Indian national survey of therapeutic drug monitoring with anti-tumor necrosis (TNF) medications in inflammatory bowel disease
Source: Indian J Gastroenterol. 2020 Jun 1;39(2):176–85. doi: 10.1007/s12664-020-01047-6 (PMC7297832; doi:10.1007/s12664-020-01047-6)
Supplement: Supplementary file 1 — (PDF 57 kb) [file 12664_2020_1047_MOESM1_ESM.pdf]

TDM Questionnaire for India

Q1. Please tell us where do you work? (Tick all that apply)

A) Government Medical college
B) Private Medical college
C) Private group practice/ Corporate hospital
D) Private individual practice

Q2. Please tell us which city do you practice in :

A) Tier 1 (Metropolitan)
B) Tier 2 (Other state capitals)
C) Tier 3 (all other cities)

Q3. Please tell us your grade:

A) Consultant – gastroenterologist
B) Physician with special interest in GI
C) Gastroenterology trainee
D) Other (please specify)

Q4. What is your gender?

A) Male
B) Female

Q5. What is your age?

A) 25-34 years
B) 35-44 years
C) 46-54 years
D) 55-64 years
E) ≥65 years

Q6. How many years (after your specialist certification) have you been in practice?

A) 0(still in training)
B) <1 year
C) 1-4 years
D) 5-9 years
E) 10-19 years
F) >20 years

Q7. What percentage of your patients have inflammatory bowel disease (IBD)?

A) <10%

B) 11-25%
C) 26-50%
D) >50%

Q8. How many patients with IBD do you evaluate and/or treat on average in a one-month period? (Please provide your best estimate. If <5: Thank you for your participation. This survey refers exclusively to medical practitioners who evaluate and/or treat >=5 IBD patients on average in a one month period)

A) <5
B) 5-10
C) 11-20
D) 20-30
E) >30

Q9. In any given month, on average how many of your IBD patients are treated with anti-TNF therapy? (Please provide your best estimate. If 0: Thank you for your participation. This survey refers exclusively to medical practioners who use anti-TNF therapy to treat IBD)

A) 0
B) 1-4
C) 5-10
D) 11-20

Q10. Do you check anti-TNF drug concentrations and anti-drug antibodies? (If no, go to question 13)

A) Yes
B) NO

Q11. In which clinical scenarios do you check anti-TNF drug concentrations to your IBD patients? (You can check more than one)

A) Secondary loss of response (patients who initially responded to anti-TNF therapy but now develop symptoms of active IBD)
B) Primary nonresponse (post-induction)
C) Re-starting anti-TNF therapy after a drug holiday
D) Proactive therapeutic drug monitoring (during maintenance therapy in patients responding to anti-TNF therapy)

Q12. How often do you check anti-TNF drug concentrations during maintenance therapy in patients responding to anti-TNF therapy (proactive therapeutic drug monitoring)?

A) Never
B) Every 2 months
C) Every 6 months
D) Every 12 months

E) Every 24 months
F) ≥24 months

Q13. Indicate how much you agree or disagree with the following statements regarding barriers to the implementation of therapeutic drug monitoring (TDM) of anti-TNF therapy in real-life clinical practice.

	Strongly disagree	Somewhat disagree	Neither agree nor disagree	Somewhat agree	Strongly agree
Test is expensive					
Uncertainty about availability in my practice					
Lack of overall knowledge of TDM					
Lack of knowledge on how to interpret and what to do with the results of TDM					
Time lag from serum sampling to results of TDM					
TDM is cumbersome and/or time consuming					
Lack of good evidence-based medicine of the usefulness of TDM in IBD					
Lack of clinical guidelines recommending the use of TDM					

Q14. If all barriers were removed, would you check anti-TNF drug concentrations? (Only for participants who replied no to question 10. If yes, go to question 16/If no, end of survey, thank you very much for your participation)

A) Yes
B) No

Q15. If all barriers were removed, in which clinical scenarios would you check anti-TNF drug concentrations to your IBD patients? (You can check more than one)

A) Secondary loss of response (patients on anti-TNF who had responded but now develop symptoms of active IBD
B) Primary nonresponse (post-induction)
C) Re-starting anti-TNF therapy after a drug holiday
D) Proactive therapeutic drug monitoring (during maintenance therapy in patients responding to anti-TNF therapy)

Q16. If all barriers were removed, how often would you check anti-TNF drug concentrations during maintenance therapy in patients responding to anti-TNF therapy (proactive therapeutic drug monitoring)?

A) Never
B) Every 2 months
C) Every 6 months
D) Every 12 months
E) Every 24 months
F) ≥24 months
